# 

**Published:** 1917-06-16

**Authors:** 


### Personalia.

Dr. Spurrell, medical superintendent of the Poplar and Stepney Sick Asylum, is making a good recovery from his severe attack of tonsilitis. By the time this appears in print he will probably be convalescing out of London. The managers of the institution have sent Dr. Spurrell a letter of sympathy.

Lieutenant Walwyn Trerice Adams, East Yorks Regiment, elder son of the Rev. S. Trerice Adams, M.A., Vicar of Holy Sepulchre Church, Cambridge, and late Chaplain of Addenbrooke's Hospital, Cambridge, has been awarded the Military Cross for distinguished service in the field.

### HOSPITAL AND INSTITUTIONAL RESULTS.

**National Society for Epileptics.**—At the end of 1916 this Society had under its care at the Chalfont Colony 338 regular colonists and eighteen convalescent or temporary patients. The weekly *per capita* cost was just over 12s., being about 1s. higher than in 1913. After negotiations with the War Pensions Statutory Committee, homes for discharged sailors and soldiers suffering from epilepsy have been established. The farm department showed a profit on the year of £823. Total income, £11,748; total expenditure, £10,857.

**East London Hospital for Children.**—The board of management announces its belief in the usefulness of the annual report in propaganda work, and has not curtailed the present issue in any way. The success of the recent appeal, helped by Mr. Punch, has extinguished the overdraft of £3,156 and provided a balance in hand for this year's working of £3,129.

**North Ormesby Hospital.**—The hospital was established, as the first cottage hospital in England, at Dundas Mews in 1858, was removed to its present site in 1861, and now contains 116 beds. There are over sixty members of the house committee, mostly representing the firms and their employees in the neighbourhood Total income, £9,001; total expenditure, £8,281.

**Bristol General Hospital.**—The report is in its customary form, and contains 142 pages. The daily average bed occupancy was 248. We do not understand for what object the out-patient attendances, on page 21, are added to the numbers of individual out-patients. The total income, including legacies £37,299, was £53,943, and the total expenditure £24,598. The ordinary expenditure has increased by £3,482 over that of 1915, provisions alone showing an increased cost of £1,230.

**Belgrave Hospital for Children.**—Of the 686 patients admitted during 1916, 191 were the children of sailors and soldiers. Both the matron, Miss F. E. Barwell, and the secretary, Mr. Clapham, are away on active service. The income last year was £4,573, and the expenditure £4,954. There were no legacies received.

**Walsall and District Hospital.**—A note of distinction is given to the report by the admirable photograph, reproduced on the cover, of the statue of Sister Dora, a "lady who devoted herself so faithfully to alleviating the condition of the sick poor of the town," and who undertook the nursing superintendence of the hospital from 1865 until she died a few weeks after the opening of the present building. The total income, including that part of extraordinary income that it is the custom to use for current expenses, was £7,068, and the total expenditure £7,662.

**Royal Berkshire Hospital, Reading.**—Whilst income has remained about stationary, the ordinary expenditure of the hospital has increased greatly. The extra cost of provisions last year was £1,521, and of salaries and wages £949. The result of this condition is that the debt on the general fund has gradually increased, until it is now £10,655.

**St. Bartholomew's Hospital, London.**—During 1916 1,309 sick or wounded soldiers were admitted, making a total of 3,335 since the commencement of the war. The total income for last year was £106,266, and the total expenditure £110,405; there has been a net deficiency of £1,049 on the working of the last three years. The cost of maintenance has risen from £86,609 in 1914 to £99,437 in 1916; under the heading of provisions the increased cost in this period was over £9,000. Thirty-four St. Bartholomew's nurses have received the Royal Red Cross and twenty-nine have been mentioned in dispatches. There are nine members of the civil staff and forty-five other employees serving with H.M. Forces.

**London Homœopathic Hospital.**—The report of the board opens with congratulations to the secretary of the hospital, Major E. A. Attwood, on his recent promotion. Seventy-five beds reserved for the use of wounded naval cases have been constantly in use. The ordinary income has increased from £9,685 in 1912 to £14,127 in 1916; in the same period expenditure has risen from £12,920 to £16,394. The income as stated includes legacies under £105 amounting to £903, but in addition larger legacies to the extent of £1,393 were received.

**Westminster Hospital.**—The report and accounts as issued to the governors are abbreviated, but a full copy, with a list of individual contributions, etc., can be seen at the secretary's office. 1,929 patients were admitted during the year, 1,525 of whom were civilians and 424 sick and wounded soldiers. The total income, including £7,455 legacies, was £27,472, and the total expenditure £28,265. The cost of provisions was £1,967 more than in 1915.

**National Hospital for the Paralysed and Epileptic.**—The report is published in full. The average weekly cost per occupied bed was lower than in 1915, although the gross expenditure was greater. There was an adverse balance on the year's working of £946. The hospital makes a speciality of accepting donations on which life annuities are paid.

RATES OF SUBSCRIPTION: Twelve months, 6/6; Six months, 4s.; three months, 2/-, post free to any address in the United Kingdom. Abroad, Twelve months, 8/8; Six months, 5/-; Three months, 2/6, post free. THE HOSPITAL "Index" to Volume LXI., October 1916 to March 1917, is now ready, price 2½d. per copy, post free. Address The Manager, THE HOSPITAL, "The Hospital" Building, 28/29 Southampton Street, Strand, London, W.C. 2.

